# Rheolytic thrombectomy using an AngioJet ZelanteDVT catheter or a Solent Omni catheter for patients with proximal vein thrombosis

**DOI:** 10.1186/s12959-023-00472-9

**Published:** 2023-03-10

**Authors:** Maofeng Gong, Guanqi Fu, Zhengli Liu, Yangyi Zhou, Jie Kong, Boxiang Zhao, Wensheng Lou, Jianping Gu, Xu He

**Affiliations:** Department of Vascular and Interventional Radiology, Nanjing First Hospital, Nanjing Medical University, Nanjing, Jiangsu 210006 People’s Republic of China

**Keywords:** Deep vein thrombosis, Percutaneous mechanical thrombectomy, AngioJet, ZelanteDVT catheter, Radiology, interventional

## Abstract

**Purpose:**

The present study aimed to investigate the preliminary safety and efficacy of rheolytic thrombectomy (RT) using an AngioJet Zelante DVT catheter or a Solent Omni catheter for acute proximal deep vein thrombosis (DVT).

**Material and methods:**

We conducted a retrospective review of 40 patients who were treated with an AngioJet RT between January 2019 and January 2021, and then the patients were divided into the ZelanteDVT group (*n* = 17) and the Solent group (*n* = 23). Data on demographics, clinical characteristics, technical success, clinical success, complications, and early follow-up were analysed.

**Results:**

No significant differences regarding demographics were detected (all *p* > .05). The technical success rates were both 100%. The ZelanteDVT group had a shorter duration of RT and a higher primary RT success than the Solent group (all *p* < .05), and the percentage of adjunctive catheter-directed thrombolysis (CDT) was 29.4% in the ZelanteDVT group, which was significantly lower than the 73.9% in the Solent group (*p* = .010). The clinical success rates for the ZelanteDVT group and Solent group were 100% (17/17) and 95.7% (22/23), respectively, and these values were high in the two groups (*p* > .05). Apart from transient macroscopic haemoglobinuria occurring in all the patients during the first 24 hours post-RT, none of the patients in either group suffered other procedure-related adverse events or major complications. Minor complications included bleeding events in 21.7% (5/23) of the patients in the Solent group and one (5.9%) patient in the ZelanteDVT group (*p* > .05). At 6 months, the frequency of PTS was 5.9% (1/17) in the ZelanteDVT group and 17.4% (4/23) in the Solent group (*p* > .05).

**Conclusion:**

Both catheters are safe and effective in managing patients with proximal DVT, thus leading to improved clinical outcomes with few complications. The ZelanteDVT catheter was more effective than the Solent catheter in thrombectomy, thus allowing for faster extraction of the DVT with a shorter run time and lower proportions of patients with adjunctive CDT.

**Supplementary Information:**

The online version contains supplementary material available at 10.1186/s12959-023-00472-9.

## Introduction

Acute proximal deep vein thrombosis (DVT), which refers to a thrombosis that emerges in the iliofemoral vein and/or popliteal vein, occurs in 1/1000 of adults annually and has become the third most common vascular disease [1–3]. Without prompt treatment, there is a high risk that DVT will develop pulmonary embolism (PE) sequelae, which has an increased risk of mortality. In recent years, many strategies have been improved and widely applied for the treatment of proximal DVT [2, 3]. Traditionally, conservative therapies mainly consist of anticoagulation and compression stockings; however, approximately half of these patients develop venous dysfunction that results in postthrombotic syndrome (PTS) [1–4]. Gradually, more aggressive endovascular therapies, including catheter-directed thrombolysis (CDT) [4], percutaneous mechanical thrombectomy (PMT) [1–3], percutaneous transluminal angioplasty (PTA) and stent placement, have emerged and these modalities have advantages compared to conservative anticoagulation therapies.

PMT, using various devices for DVT, has become an option that be quickly performed in addition to open surgical thrombectomy [2, 3]. Mechanisms of thrombus removal mainly include suction, rotation, rheolytic thrombectomy (RT) and ultrasound [1]. The AngioJet device, which represents a main modality for the treatment of RT, is based on a pharmacomechanical thrombectomy via active aspiration and pulsatile lytic delivery [1, 5]. It can facilitate the rapid removal of a thrombus, potentially decrease the recurrence of DVT, and further reduce the severity of PTS [2, 3, 5]. However, the main outcomes underlying different specifications of commonly applied AngioJet catheters remain unclear. More than half of the PEARL patients who received a single Solent® catheter experienced incomplete thrombus removal, and adjunctive CDT was needed. The ZelanteDVT® catheter (8-French (F), Boston Scientific, Maple Grove, MN, USA) is a novel RT catheter that has been inserted for DVT treatment in recent years [6, 7]. However, relatively few studies on the performance and complications of the two catheters have been reported.

Dopheide et al. [6] first reported the utility of a ZelanteDVT catheter for the treatment of proximal DVT. The ZelanteDVT, a novel RT catheter, has been updated and exhibits the following features in comparison to the Solent catheter: a) an 8-F catheter lumen which is larger than a 6-F lumen, b) a modified structure at the treatment segment that has one larger proximal suction window and one smaller distal outflow window, and c) a rotational hub to achieve directional thrombus removal [6, 7]. Although two AngioJet models using different catheters use the same mechanistic platform, whether this novel ZelanteDVT catheter has improved safety and competitive catheter performance when compared to the Solent catheter in clinical practice remains obscure because there is a lack of a comparative study [2]. Better knowledge of these catheter characteristics might lead to optimized differentiated selection and therefore lead to a reduction in complications and improved long-term venous patency.

The purpose of the present study was to compare the preliminary outcomes, including grade of venous thrombus removal, clinical outcomes, and potential complications, of the AngioJet ZelanteDVT catheter and the conventional Solent Omni catheter (6-F, Boston Scientific, Fremont, Calif, USA) in patients with acute proximal DVT.

## Materials and methods

### Study design and patients

The Institutional Review Board of our hospital approved this retrospective single-centre study and waived the requirement for written informed consent for the use of electronic medical records and imaging data. Informed consent was obtained from all participating patients receiving endovascular treatment before therapy. Before August 2019, the AngioJet ZelanteDVT catheter was not an available option in our country. The Solent catheter was the exclusive catheter of choice. The ZelanteDVT catheter has been a choice since August 2019, when it became available.

From January 2019 to January 2021, 40 consecutive patients (mean age 58.9 ± 18.5 years; 65% female) with proximal DVT involving the popliteal, femoral, common femoral, and/or iliac veins (with or without other involved ipsilateral veins) who underwent AngioJet RT using a ZelanteDVT catheter (ZelanteDVT group) or a Solent catheter (Solent group) were recruited. RT was performed by two senior endovascular operators with > 10 years of extensive experience in endovascular therapy in both groups. All the patients were treated by RT with the same type of AngioJet pump unit (Boston Scientific, Fremont, Calif, USA). Patients were included in this study if they met the following inclusion criteria: diagnosed with acute phase (had symptom onset < 14 days) and with at least 180 days of follow-up; experienced proximal DVT; and a ZelanteDVT or Solent catheter was inserted for RT. The exclusion criteria for the included patients were age < 18 years or estimated life expectancy < 3 months.

### Management strategies

The initial diagnosis of proximal DVT in each treatment group was verified by medical history and physical examination and then objectively confirmed by compression ultrasound. If these results were inconclusive, supplementary venography was performed. When DVT was identified, management strategies were instantly performed. The option of an AngioJet Zelante DVT catheter or a Solent catheter was left to the discretion of the group of endovascular operators, and mainly depended on the catheter availability and clinical experience.

#### AngioJet RT reperfusion

Following popliteal access (required with the use of ultrasound guidance) or femoral access with a 10-F sheath under local anaesthesia and strict sterile techniques, RT using a ZelanteDVT catheter or a Solent catheter was performed for pharmacomechanical thrombus fragmentation, suction or aspiration. First, the RT catheter was slowly advanced through the thrombotic segment (only submerged in vessel diameter estimated > 6 mm). For patients without contraindications of thrombolysis, 3 mg of rt-PA [total injected volume of 50 ml] was intraclot injected under the Power Pulse® model. After 20 minutes of dwell time, with the pump unit active during slow catheter passages (3 mm/s to 5 mm/s), runs were performed across the thrombotic segment in a distal-to-proximal or adverse direction under fluoroscopic guidance. Each device activation run lasted at less than 20 seconds with breaks of 30 seconds between the runs to avoid arrhythmia, and the total run times were monitored and kept no more than 240 seconds.

#### Adjunctive with reduced-dose CDT

If residual thrombus (defined as thrombus removal grade ≤ I) was present and did not meet the exclusion criterion of thrombolytic contraindications, a continuous infusion of reduced-dose recombinant tissue plasminogen activator (rt-PA) (Alteplase; Boehringer-Ingelheim, Ingelheim am Rhein, Germany) was delivered subsequently via a multi-side hole catheter (Uni*Fuse, AngioDynamics, Boston Scientific, USA) embedding into the thrombus. Then, 17 mg/20 mg alteplase was administered at an infusion rate of 0.01 mg/kg per hour following CDT. The maximum rate was less than 1.0 mg/h, and the total doses were less than 50 mg, as noted elsewhere [8]. CDT was discontinued when at least 80% clot lysis with restoration of flow or a serious complication occurred. Alteplase was administered only when the fibrinogen level was > 1.0 g/L.

#### Other comprehensive therapy

A temporary filter was inserted via the nonaffected femoral or jugular vein into the inferior vena cava (IVC) prior to the next procedure for patients with an extensive thrombus in the proximal vein that was evaluated as potentially life-threatening and was retrieved after the proximal DVT was removed and potentially life-threatening conditions were relieved. Consistent with local routines based on published guidelines [9], anticoagulant treatment was initiated immediately when DVT was identified with the use of subcutaneous low molecular weight heparin (LMWH) at a bolus dose of 100 units/kg twice daily. PTA and/or stent placement was encouraged for lesions that caused 50% or greater diameter narrowing of the iliac and/or common femoral vein, robust collateral filling, and/or a mean pressure gradient of more than 2 mmHg. At the end of LMWH, oral rivaroxaban was directly commenced at a dosage of 15 mg twice a day over the subsequent 21 days and 20 mg once a day thereafter for at least 6 months. In addition, the use of compression stockings (ankle pressure was approximately 30–40 mmHg) for more than 1 year was recommended.

### Outcomes and safety

Technical success was defined as the successful use of AngioJet RT. Thrombus score was calculated through venography imaging by two experienced interventional physicians independently depending on pre-RT, at the completion of RT or post-CDT, by adding the scores of six vein segments (common iliac vein, external iliac vein, common femoral vein, proximal and distal segments of femoral vein, and popliteal vein). Thrombus scores were 0 when the vein was patent and completely free of thrombus, 1 in condition of a partially occluded vein, and 2 in condition of a completely occluded vein (i.e., vein lumen completely occluded with massive thrombus). The score was calculated for each segment, resulting in possible total thrombus scores. The thrombus removal rate was calculated as follows: [total pre-RT scores - total completion of RT (or total post-CDT scores)]/total pre-RT scores × 100%. Thrombus removal grades were evaluated as grade III (100% thrombus removal rate with no residual clots), grade II (50–99% thrombus removal rate), and grade I (< 50% thrombus removal rate). Thrombus removal grades II and III (i.e., ≥50% thrombus removal rate) were considered clinical success [10], which consisted of primary RT success and adjunctive CDT success. The primary RT (defined as patients who did not require adjunctive CDT treatment) success was classified based on preprocedural and at completion of RT thrombus scores evaluated as grade II and grade III. Adjunctive CDT (defined as patients who required adjunctive CDT treatment) success was classified based on preprocedural thrombus scores and those at the end of adjunctive CDT that were evaluated as grade II and grade III. The requirement of necessary adjunctive PTA and/or stent placement to treat coexisting stenosis to obtain sufficient flow within the same hospital stay was recorded but not considered clinical failure.

The safety outcomes consisted of procedure-related and CDT-related complications. The former included vessel perforation or damage (such as extravasation or retention of contrast agent in the vessel wall), bradycardia, arrhythmias or acute kidney injury (AKI). With adherence to the Society of Interventional Radiology (SIR) [11], the latter feature was divided into major CDT-related complications, which were defined as intracranial bleeding or bleeding severe enough to result in death, surgery, cessation of therapy, or blood transfusion, and minor complications, which were defined as less severe bleeding manageable with local compression, sheath upsizing, and/or alterations of thrombolytic agent dose and anticoagulant dose [11]. The SIR classification of complications is listed in the Supplementary Table.

### Follow-up

The data on the resolution of symptoms were evaluated at the 6-month follow-up via re-examination or telephone using the Villalta PTS Scale [12], which is scored on a 4-point scale (0 = none, 1 = mild, 2 = moderate, 3 = severe). Short-term outcomes were defined as data at the 6-month follow-up, including recurrent DVT, hospital readmission, or death.

### Statistical analysis

The *SPSS* statistical software package (version 23.0; *SPSS* statistical software, Chicago, Illinois, USA) was used to perform all statistical analyses in this study. Continuous variables are expressed as the mean ± standard deviation. Here, *t tests* were used to assess the correlation between preprocedural and postprocedural variables and between groups. Qualitative variables are presented as numbers and percentages. The significance of qualitative variables was assessed using *Fisher’s* exact test. Findings with a *p* value less than 0.05 were deemed statistically significant.

## Results

### Baseline demographics and clinical characteristics

A total of 40 patients were included in this study, with 17 patients in the ZelanteDVT group and 23 patients in the Solent group. The demographics and characteristics of these 40 patients are summarized in Table 1. No significant differences regarding age, sex, onset time, limb characteristics, or risk factors were detected between the two groups (all *p* > .05).

**Table 1:** Patients’ Demographics, and Presentation, Lesion Characteristics and Risk Factors of DVT

Characteristic	ZelanteDVT Group (*n* = 17)	Solent Group (*n* = 23)
Age, years, mean ± SD	55.4 ± 21.5	60.4 ± 15.4
Female, sex, n (%)	10 (58.8)	16 (69.6)
Onset of Symptoms at Presentation, n (%)		
≤ 7 days	11 (64.7)	16 (69.6)
≥ 7 days and ≤ 14 days	6 (35.3)	7 (30.4)
Thrombus Segment		
Isolated iliofemoral DVT	11 (64.7)	15 (65.2)
With popliteal/calf DVT	6 (35.3)	8 (34.8)
Thrombus Limbs		
Left	9 (52.9)	16 (69.6)
Right	8 (47.1)	7 (30.4)
Bilateral	0 (0)	0 (0)
Risk Factors, n, (%)		
Major surgery history	3 (17.6)	5 (21.7)
Immobilization	2 (11.8)	3 (13.0)
Hospitalization	2 (11.8)	4 (17.4)
Recent trauma	4 (23.5)	3 (13.0)
May–Thurner syndrome	1 (5.9)	4 (17.4)
Childbirth	1 (5.9)	1 (4.3)
Malignant disease history	1 (5.9)	3 (13.0)
Autoimmune diseases	1 (5.9)	2 (8.7)
Previous DVT or PE	4 (23.5)	3 (13.0)
Family history of venous thromboembolism	1 (5.9)	2 (8.7)
Unknown factors	4 (23.5)	4 (17.4)

*DVT* Deep vein thrombosis, *PE* Pulmonary embolism

Continuous data are presented as the means ± standard deviations; categorical data are given as the counts (percentage)

### Procedure details and outcomes

All the patients in the two groups received recoverable IVC filters prior to RT treatment, and all filters were successfully retrieved, filling defects into the filters were observed in 5.0% (2/40) patients during repeat venography, which disappeared after thrombolysis. All patients underwent AngioJet RT successfully, and the technical success rates were both 100%. The intraoperative procedures and treatment details are shown in Table 2.

**Table 2:** Procedure Characteristics by Treatment Catheters and Outcomes

Characteristics	ZelanteDVT Group (*n* = 17)	Solent Group (*n* = 23)	*p value*
Success of thrombus removal, n (%)			
Primary RT success	12 (70.6)	6 (26.1)	.010
Adjuvant CDT success	5 (29.4)	16 (69.6)	.018
Clinical success	17 (100)	22 (95.7)	1.000
Adjuvant other endovascular methods, n (%)			
Balloon angioplasty	11 (64.7)	13 (56.5)	.747
Stent placement	6 (35.3)	4 (17.4)	.274
Duration of operation procedure, (h, mean ± SD)	1.61 ± .15	1.72 ± .22	.086
RT procedure			
Patients used PP model, n (%)	14 (82.4)	20 (87.0)	1.000
Mean RT duration time, (s, mean ± SD)	47.65 ± 14.37	73.91 ± 18.52	.000
Adjuvant CDT procedure			
Number, n (%)	5 (29.4)	17 (73.9)	.010
Mean duration time, d	2.00 ± 1.22	3.00 ± 1.27	.136
rt-PA dose, mg	18.20 ± 11.10	27.35 ± 12.19	.149
Thrombus scores			
Pre-RT	9.65 ± 2.15	9.57 ± 1.90	.899
RT-completion	2.71 ± 2.64	4.35 ± 2.31	.043
CDT-completion	2.00 ± 1.40	1.65 ± 1.46	.637
Thrombus removal grades, n (%)			
Grade I	0 (0)	1 (4.3)	1.000
Grade II	10 (58.8)	13 (56.5)	1.000
Grade III	7 (41.2)	9 (39.1)	1.000
Complications, n (%)			
Minor (SIR A, B: nominal or no therapy, no consequence)	1 (5.9)	5 (21.7)	.216
Major (SIR C, D, E: requires therapy or permanent sequelae)	0 (0)	0 (0)	–
Major-death (SIR F: death)	0 (0)	0 (0)	–
Procedure-related PE, n (%)	0 (0)	0 (0)	–
In-hospital length of stay, d	7.99 ± 2.09	9.91 ± 3.00	.030
PTS at 6-month follow-up, n (%)	1 (5.9)	4 (17.4)	.546

*DVT* Deep vein thrombosis, *CDT* Catheter-directed thrombolysis, *RT* Rheolytic thrombectomy, *PP* Power pulse

Continuous data are presented as the means ± standard deviations; categorical data are given as the counts (percentages)

No significant difference in the mean procedural time was noted between the two groups (1.61 ± .15 h vs. 1.72 ± .22 h; *p* > .05), but the ZelanteDVT group had a shorter RT duration (47.65 ± 14.37 s vs. 73.91 ± 18.52 s; *p* = .000) (listed in Table 2). As calculated, the thrombus scores pre-RT in the ZelanteDVT group and Solent group were similar (9.65 ± 2.15 vs. 9.57 ± 1.90; *p* > .05). After the RT procedure, the thrombus scores of the ZelanteDVT group significantly decreased (9.65 ± 2.15 vs. 2.71 ± 2.64; *p* < .05). The primary RT success was more apparent in the ZelanteDVT group than in the Solent group (70.6% vs. 26.1%; *p* = .010). The percentage of patients who needed adjunctive CDT was significantly lower in the ZelanteDVT group than in the Solent group (29.4% vs. 73.9%; *p* = .010). The total adjunctive CDT time and agent dosages were not significantly different (all *p* > .05). The ultimate thrombus scores and thrombus removal grades at the end of adjunctive CDT in the two groups were similar. The ZelanteDVT group had a shorter hospitalization time than the Solent group (7.99 ± 2.09 d vs. 9.91 ± 3.00 d; *p* = .030).

At completion, 64.7% (11/17) of the patients in the ZelanteDVT group underwent PTA, and 56.5% (13/23) of the patients in the Solent group underwent PTA. The 35.3% (6/17) rate of stent use in the ZelanteDVT group seemed to be slightly higher than that in the Solent group (17.4%, 4/23), but the difference was not significant (*p* > .05). At the time of discharge, the clinical success rates for the ZelanteDVT group and the Solent group were 100% (17/17) and 95.7% (22/23), respectively. These success rates were high in both groups but the difference was not statistically significant (*p* > .05). The use of antithrombotic and compression treatments was not significantly different between the two groups (*p* > .05). At 6 months, the frequency of PTS was 5.9% (1/17) in the ZelanteDVT group compared with 17.4% (4/23) in the Solent group. All patients presented with mild PTS, and none had moderate or severe PTS.

### Complications

Except for transient macroscopic haemoglobinuria occurring during the first 24 hours post-RT in all patients (40/40), no other procedure-related adverse events, including arrhythmia, symptomatic/fatal PE, and AKI, were recorded in either group. The creatinine clearance rate and blood urea nitrogen levels in the ZelanteDVT group (69.9 ± 27.1 μmol/L and 5.0 ± 1.9 mmol/L, respectively) were slightly higher than those in the Solent group (68.2 ± 20.7 μmol/L and 4.5 ± 2.0 mmol/L, respectively) but without statistical significance (both *p* > .05). No major adjunctive CDT-related complications were noted in either group (the rates of major complications, including SIR categories C, D, E, and F, were all 0%). In the Solent group, minor complications such as bleeding events (SIR B category) occurred in 21.7% (5/23) of patients, including two small haematomas at the access site, two superficial ecchymoses, and one epistaxis. A total of 5.9% (1/17) of the patients in the ZelanteDVT group had superficial haematomas (SIR B category), and the difference was not statistically significant (*p* > .05). All patients recovered with conservative treatment, and no life-threatening bleeding events, recurrent DVT, or 30-day death were observed.

## Discussion

The present study found that the clinical success rates for the ZelanteDVT group and Solent group were high, which indicated that both catheters were effective in managing patients with proximal DVT. However, the ZelanteDVT group had a higher primary RT success, which means that proximal vein patency was rapidly restored and significantly less patients needed adjunctive CDT (29.4%) than the Solent group (73.9%) to restore venous patency. The reduced requirement for sequential CDT findings also indicated that ZelanteDVT seems to have a shorter hospitalization time, lower total dose of rt-PA, and reduced incidence of bleeding complications; however, the latter two factors did not differ significantly in the present study. Regarding increased primary RT success without the need for prolonged adjunctive CDT and a shorter RT duration in the ZelanteDVT group, these outcomes were previously noted in the study published by Kwon et al. [13], who reported more effective remodelling of a large thrombus due to the more powerful negative pressure. These features may likely be attributed to the mechanistic differences between the catheters considering that this novel catheter provides a stronger biological suction effect (− 600 mmHg) and has a large lumen at the tip of the catheter. As well, over the wire suction or rheolytic devices seem to have better results with larger diameter devices since the catheters can’t be directed to the vein wall.

A limited number of RCT studies, such as the ATTRACT trial [3] and a multicentre PEARL trial [5], reported that the Solent strategy was used to remove > 50% of thrombi (grade II and grade III) in more than 95% of treated patients. Additionally, Dopheide et al. reported a relatively high thrombus removal rate for ZelanteDVT [8]. However, these studies mentioned above were limited because they did not provide comparisons between the ZelanteDVT catheter and the Solent catheter. Our present work showed that although the > 50% thrombus removal rates were similar for both catheters, the exclusive use of the ZelanteDVT catheter achieved rapid restoration of blood flow. Although the frequency of PTS in the ZelanteDVT group (5.9%) was not significantly superior to that in the Solent group (17.4%) during the early 6-month follow-up period, earlier restoration of blood flow will reduce thrombus burden and vein wall fibrosis, which may reduce the clinical incidence of PTS [3, 5, 14]; hence, we hypothesized that the ZelanteDVT catheter may achieve promising long-term efficacy. However, the present study was limited because the long-term follow-up was not assessed, and further confirmation of the conclusions is needed.

A range of adverse events was plausibly associated with the use of AngioJet [2–4]. In the PEARL trial, AngioJet-related events, such as PE, renal failure, low haemoglobin, and bradycardia, were recorded [5]. In this study, except for the occurrence of transient macroscopic haemoglobinuria in both groups in the first 24 hours postintervention, no other side effects of bradycardia or low haemoglobin were recorded. AKI is a well-known complication of RT and is probably due to haemolysis caused by the high-pressure saline jets of the RT system as well as the nephrotoxic contrast agents used for venography [2]. Regarding the mechanisms of AngioJet RT, the ZelanteDVT catheter posed a stronger suction effect, which seems to have a higher potential risk of AKI. However, patients in the present study with severe renal dysfunction were excluded, and there were limits on RT activation time, aspiration volume, and preprocedural hydration. The indices of kidney function did not differ significantly. Haemorrhagic complications are likely associated with rt-PA and anticoagulation treatments [2]; they were less frequently noted in ATTRACT when compared with other large CDT studies, perhaps due to the reduced rt-PA dose and infusion times [2–4]. As in the present study, due to the reduced use of adjunctive CDT, the ZelanteDVT catheter reduced the risk of bleeding complications when compared to the Solent catheter. Safety may be further improved if future refinements can support the exclusive and frequent use of AngioJet RT.

The routine use of IVC filters remains controversial for patients undergoing PMT or CDT. Lee et al. [15] demonstrated that the filter should be considered for preventing PE-related morbidity. The incidence of symptomatic and fatal PE in patients who received CDT only treatment was 0–1.3% and 0–0.2%, respectively [2–5], but the rates increased when endovascular procedures were performed [2–4, 16]. Hence, mechanical endovascular procedures seem to increase the risk of PE events, which may be associated with multiple risk factors, such as nonblocking iliac veins, massive proximal floating thrombi, and invasive endovascular procedures. In the present study, all patients underwent routine IVC filter insertion; as a result, some filling defects into the filters were observed during repeat venography, which disappeared after thrombolysis. No PE-related events were recorded during the procedure or adjunctive CDT. Iliac vein stenosis is one of the anatomical factors for DVT [2]. To decrease distal venous hypertension, relieve clinical symptoms, prevent DVT recurrence, and improve the prognosis, 64.7% of patients had segmental (> 50%) residual stenosis at the end of the ZelanteDVT procedure to successfully remove a thrombus and to sufficiently restore blood inflow, this outflow obstruction was resolved by one-stage PTA and/or subsequent stent placement, which was conducted after CDT in 56.5% of the patients who received a Solent catheter, the rate of PTA was higher than in literatures [1–4]. The use of stent placement in the ZelanteDVT group was slightly higher than that in the Solent group. This finding was associated with slightly increased thrombus removal, which ensured sufficient blood inflow passages and created a condition for stent placement. Of noted, the percentage of stenting in both groups is lower than generally described, which may be partly attributed to the lower residual stenosis after PTA and higher thrombus removal.

Our study had several limitations. First, the present study used a fixed-length catheter (105 cm of ZelanteDVT and 120 cm of Solent Omni) designed for AngioJet RT; thus, whether the length would affect the RT technique was difficult to evaluate. The diameters of the deep veins may have also affected the results, and this observance should be further evaluated in future studies. Second, different grades of catheters, other than 6F and 8F, were not used because catheters with other diameters were unavailable at the time. Third, the use of different catheters was not random but was mainly based on catheter availability and clinical experience at our centre in different phases, which may have caused selection bias regarding the evaluation of the outcomes of each catheter. Fourth, this study was limited to a short follow-up period of 6 months, and long-term outcomes will be recorded at 1 year. Fifth, the complications were not completely classified according to the standard of SIR because almost all the complications occurred during hospitalization, and we primarily categorized these complications based on which therapy was required and the prognosis. Moreover, the present study was a single-centre, retrospective, nonrandomized controlled study. The conclusions of the present study were limited because of the small number of cases and limited operator experience and should be interpreted with caution. In the future, a prospective, randomized controlled study with a longer follow-up period to overcome these limitations is needed to further confirm the conclusions.

## Conclusion

The present study found that both catheters are safe and effective in managing patients with proximal DVT, thus leading to improved outcomes with a low complication rate. The ZelanteDVT catheter is more effective than the previous Solent catheter in thrombectomy, thus allowing for faster extraction of the DVT with a shorter run time, lower proportions of patients needing adjunctive CDT, increased primary thrombus removal, and a shorter hospitalization time. The ZelanteDVT catheter seems to be a better purpose-built catheter that allows for greater ease in the extraction of proximal vein thrombi.

## Supplementary Information

**Additional file 1: Supplementary Table.** SIR Classification of Bleeding Complications.

## Data Availability

The datasets generated and analysed during the current study are not publicly available, as the experimental data are related to other experiments that are progressing but are available from the corresponding author upon reasonable request.

## References

[1] Thukral S, Vedantham S (2020). Catheter-based therapies and other management strategies for deep vein thrombosis and post-thrombotic syndrome. J Clin Med.

[2] Vedantham S, Salter A, Lancia S, Lewis L, Thukral S, Kahn SR (2021). Clinical outcomes of a pharmacomechanical catheter-directed venous thrombolysis strategy that included rheolytic thrombectomy in a multicenter randomized trial. J Vasc Interv Radiol.

[3] Vedantham S, Goldhaber SZ, Julian JA, Kahn SR, Jaff MR, Cohen DJ, Magnuson E, Razavi MK, Comerota AJ, Gornik HL, Murphy TP, Lewis L, Duncan JR, Nieters P, Derfler MC, Filion M, Gu CS, Kee S, Schneider J, Saad N, Blinder M, Moll S, Sacks D, Lin J, Rundback J, Garcia M, Razdan R, VanderWoude E, Marques V, Kearon C (2017). Pharmacomechanical catheter-directed thrombolysis for deep-vein thrombosis. N Engl J Med.

[4] Haig Y, Enden T, Grøtta O, Kløw NE, Slagsvold CE, Ghanima W, Sandvik L, Hafsahl G, Holme PA, Holmen LO, Njaaastad AM, Sandbæk G, Sandset PM (2016). Post-thrombotic syndrome after catheter-directed thrombolysis for deep vein thrombosis (CaVenT):5-year follow-up results of an open-label, randomised controlled trial. Lancet Haematol.

[5] Garcia MJ, Lookstein R, Malhotra R, Amin A, Blitz LR, Leung DA, Simoni EJ, Soukas PA (2015). Endovascular management of deep vein thrombosis with rheolytic thrombectomy: final report of the prospective multicenter PEARL (peripheral use of AngioJet Rheolytic Thrombectomy with a variety of catheter lengths) registry. J Vasc Interv Radiol.

[6] Dopheide JF, Sebastian T, Engelberger RP, Haine A, Kucher N (2018). Early clinical outcomes of a novel rheolytic directional thrombectomy technique for patients with iliofemoral deep vein thrombosis. Vasa.

[7] Ni Q, Long J, Guo X, Yang S, Meng X, Zhang L, Fang X, Ye M (2021). Clinical efficacy of one-stage thrombus removal via contralateral femoral and ipsilateral tibial venous access for pharmacomechanical thrombectomy in entire-limb acute deep vein thrombosis: a retrospective cohort study. J Vasc Surg Venous Lymphat Disord.

[8] Gong M, Zhao B, He X, Gu J, Chen G (2019). Feasibility of low-dose infusion of alteplase for unsuccessful thrombolysis with urokinase in deep venous thrombosis. Exp Ther Med.

[9] Kakkos SK, Gohel M, Baekgaard N, Bauersachs R, Bellmunt-Montoya S, Black SA, Ten Cate-Hoek AJ, Elalamy I, Enzmann FK, Geroulakos G, Gottsäter A, Hunt BJ, Mansilha A, Nicolaides AN, Sandset PM, Stansby G, Esvs Guidelines Committee, de Borst GJ, Bastos Gonçalves F, Chakfé N, Hinchliffe R, Kolh P, Koncar I, Lindholt JS, Tulamo R, Twine CP, Vermassen F, Wanhainen A, Document Reviewers, De Maeseneer MG, Comerota AJ, Gloviczki P, Kruip MJHA, Monreal M, Prandoni P, Vega de Ceniga M. Editor’s Choice-European Society for Vascular Surgery (ESVS) 2021 Clinical practice guidelines on the management of venous thrombosis. Eur J Vasc Endovasc Surg 2021;61:9–82. 10.1016/j.ejvs.2020.09.023. Epub 2020 Dec 15. 10.1016/j.ejvs.2020.09.023 33334670

[10] Mewissen MW, Seabrook GR, Meissner MH, Cynamon J, Labropoulos N, Haughton SH (1999). Catheter-directed thrombolysis for lower extremity deep venous thrombosis: report of a national multicenter registry. Radiology.

[11] Vedantham S, Thorpe PE, Cardella JF, Grassi CJ, Patel NH, Ferral H, Hofmann LV, Janne d'Othée BM, Antonaci VP, Brountzos EN, Brown DB, Martin LG, Matsumoto AH, Meranze SG, Miller DL, Millward SF, Min RJ, Neithamer CD, Rajan DK, Rholl KS, Schwartzberg MS, Swan TL, Towbin RB, Wiechmann BN, Sacks D, CIRSE and SIR Standards of Practice Committees (2009). Quality improvement guidelines for the treatment of lower extremity deep vein thrombosis with use of endovascular thrombus removal. J Vasc Interv Radiol.

[12] Villalta S, Bagatella P, Piccioli A, Lensing A, Prins M, Prandoni P (1994). Assessment of validity and reproducibility of a clinical scale for the post-thrombotic syndrome. Haemostasis.

[13] Kwon SH, Ahn SE, Shin JS, Youn HC, Kim JH, Oh JH (2016). A phantom model study to identify the most effective manual aspiration thrombectomy for acute deep-vein thrombosis of the lower extremity. Clin Radiol.

[14] Li W, Kessinger CW, Orii M, Lee H, Wang L, Weinberg I, Jaff MR, Reed GL, Libby P, Tawakol A, Henke PK, Jaffer FA (2021). Time-restricted salutary effects of blood flow restoration on venous thrombosis and vein wall injury in mouse and human subjects. Circulation.

[15] Lee SH, Kim HK, Hwang JK, Kim SD, Park SC, Kim JI (2016). Efficacy of retrievable inferior vena cava filter placement in the prevention of pulmonary embolism during catheter-directed thrombectomy for proximal lower-extremity deep vein thrombosis. Ann Vasc Surg.

[16] Kwon SH, Oh JH, Seo TS, Ahn HJ, Park HC (2009). Percutaneous aspiration thrombectomy for the treatment of acute lower extremity deep vein thrombosis: is thrombolysis needed?. Clin Radiol.

