# Supplementary material for: Rheolytic thrombectomy using an AngioJet ZelanteDVT catheter or a Solent Omni catheter for patients with proximal vein thrombosis
Source: Thromb J. 2023 Mar 10;21:25. doi: 10.1186/s12959-023-00472-9 (PMC9999514; doi:10.1186/s12959-023-00472-9)
Supplement: Supplementary file 1 — Additional file 1: Supplementary Table. SIR Classification of Bleeding Complications. [file 12959_2023_472_MOESM1_ESM.docx]

T=deep vein thrombosis; CDT=catheter-directed thrombolysis; RT=rheolytic thrombectomy; PP=power pulse

Continuous data are presented as the means ± standard deviations; categorical data are given as the counts (percentages).

Supplementary Table. SIR Classification of Bleeding Complications.

| Supplementary Table. SIR Classification of Bleeding Complications. | |
| --- | --- |
| Category | Description/prognosis |
| Minor Complications |  |
| SIR A | No therapy, no consequence |
| SIR B | Nominal therapy, no consequence; includes overnight admission for observation only. |
| Major Complications |  |
| SIR C | Require therapy, minor hospitalization (<48 hours) |
| SIR D | Require major therapy, unplanned increase in level of care, prolonged hospitalization (>48 hours) |
| SIR E | Permanent adverse sequelae |
| SIR F | Death |

SIR = Society of Interventional Radiology

This is a similar replica of the table in the 2009 publication by Vedantham S et al.^11^
